# Abortion, Brain Death, and Coercion

**DOI:** 10.1007/s11673-023-10268-1

**Published:** 2023-06-28

**Authors:** Michael Nair-Collins

**Affiliations:** Florida State University College of Medicine, 1115 West Call Street, Tallahassee, FL 32304 USA

**Keywords:** Abortion, Brain death, Accommodation, Objection, Brain death conflict, End-of-life-issues

## Abstract

A “universalist” policy on brain death holds that brain death is death, and neurologic criteria for death determination are rightly applied to all, without exemptions or opt outs. This essay argues that advocates of a universalist brain death policy defend the same sort of coercive control of end-of-life decision-making as “pro-life” advocates seek to achieve for reproductive decision-making, and both are grounded in an illiberal political philosophy. Those who recognize the serious flaws of this kind of public policy with respect to abortion must apply the same logic to brain death.

## Introduction

The U.S. Supreme Court held in *Dobbs v. Jackson Women’s Health Organization* that there is no right to abortion in the Constitution of the United States, overruling its previous determinations in *Roe v. Wade* and *Planned Parenthood v. Casey* and opening the door for individual states to ban abortion. This is among the most impactful and significant rulings in the entire history of the Court. At the same time, the issue of brain death continues to draw scrutiny. Legal challenges to brain death determination mount every year (Pope 2018), and the U.S. Uniform Law Commission is in the process of drafting revisions to the Uniform Determination of Death Act, for the first time since it was promulgated in 1981 (Uniform Law Commission). Two prominent issues of sustained bioethical focus, abortion and brain death, are in a state of flux and legal uncertainty in the United States to a degree that has not occurred in forty or fifty years.

Although especially salient at this moment in the United States, these are global issues. The last quarter century has seen almost fifty countries enacting progressive reform of abortion laws. At the same time, 118 countries continue to prohibit abortion, either without exception or only to protect the health or life of the pregnant person (Center for Reproductive Rights). Joining the United States in its regression, Poland recently implemented a near-total abortion ban in 2020 (Amnesty International 2023).

Brain death is also a matter of ongoing global dispute. Brain death cases have made their way to high courts in Australia, Canada, and the United Kingdom (McGee and Gardiner 2019; Tibballs and Bhatia 2021). Many eastern nations, including China, Japan, South Korea, and Taiwan continue to demonstrate less acceptance of brain death than western countries (Yang and Miller 2015). Continuing efforts to harmonize international practices demonstrate ongoing global concern about brain death among professional groups (Shemie et al. 2014; Greer et al. 2020).

Abortion and brain death are often compared to each other. Both implicate concepts of personhood, death, autonomy, and related moral and ontological concerns. Abortion and brain death are not the same issue, of course, and their elision would grossly oversimplify both. But there is a significant overlap, recognition of which is important and helpful especially for evaluating brain death laws and practices. Specifically, the so-called “pro-life” (anti-choice) view on abortion is both coercive and grounded in an illiberal political philosophy. The dominant approach to brain death policy, which I call “universalist” in that it seeks to disallow exemptions from death determination on neurological grounds—discussed below—is similarly coercive and grounded in an illiberal political philosophy. Those who oppose abortion prohibition on the grounds of its coercion and illiberalism should apply the same logic to brain death.

## Abortion

Many factors are relevant to ethical and policy discourse about whether abortion may be justly restricted or prohibited. I won’t attempt to summarize this literature, but simply note that key questions often debated include whether (or when) the fetus is a living organism distinct from the person in whose womb it develops; when sentience and self-consciousness develop; whether it is a person; whether it has a soul; whether it has rights or an independent welfare. Answers to these questions typically lead to an evaluation of the fetus’s moral status: is it an entity properly conceived of as owed direct moral consideration for its own sake? Finally, thoughts on the above, in conjunction with consideration of the rights and welfare of the pregnant person, inform answers to these questions: What actions are permissible to take with respect to the fetus? What obligations, if any, does the pregnant person, or others, have to the fetus? Most importantly, may the pregnant person terminate their pregnancy?

Whatever answer one might arrive at with respect to the first-order moral question as to what is permissible with respect to an individual case, the second-order political question arises: What laws and policies would a just society implement?

Two different families of ideas can be presented in answer to the latter question, which are based on a political philosophy that is, broadly speaking, either *liberal* or *illiberal*. The illiberal view, associated with the pro-life movement, holds that there is a specific answer to the question about the fetus’s moral status, which is objectively and uniquely correct and should be enforced through law. That answer is commonly associated with an interpretation of Christian or natural law theology. Namely, the fetus has the same moral status as a born human, and it is impermissible to terminate a pregnancy. The illiberal aspect of this view is that *this is the unique right answer* that a just society should implement. Other views which disagree are simply mistaken, regardless of whether they are sincerely and thoughtfully held. It does not matter if the pregnant woman does not share this worldview, because this worldview is objectively correct. Furthermore, a just society would use the coercive authority of the State to force pregnant people to conform to this moral and metaphysical worldview and, potentially, enforce this conformation through punishment. The State is therefore permitted, indeed required, to coercively prohibit abortion on this illiberal view.

The liberal view holds that a government should not take a substantive position about these kinds of deep moral and metaphysical worldviews. Reasonable people can and do reasonably disagree about them and still live together in a functioning society. It is reasonable to embrace the worldview that accords equal moral status to a fetus as to a born human, thus, one should not be forced to act in ways that are not in accordance with that view. For example, people should not be forced to have abortions. But it is also reasonable to hold a moral and metaphysical worldview that entails that terminating a pregnancy is permissible, thus, people should not be forced to carry a pregnancy against their will, either. In these deep matters of metaphysics and religion, and in personal healthcare decisions, the State ought to remain neutral, allowing that conscientious decisions be made by individuals in line with their own religious and moral commitments. Hence, the decision to carry or terminate a pregnancy is properly left to the person who is pregnant. This is the standard “pro-choice” view.

In the current political climate of the United States, and elsewhere, there is much more going on with efforts to prohibit abortion than an intellectual difference about relatively abstract matters of metaphysics. Abortion prohibition reinforces patriarchal norms and hierarchical, oppressive sex and gender roles. It specifically violates the rights of women and others capable of pregnancy to make their own healthcare decisions, to control their bodies, and to control their reproduction, all of which have reverberating impacts on education, employment, health, and so on. And it will disproportionately impact people of lower socioeconomic status, people of colour, and, with especial cruelty, survivors of sexual assault including children.

Framing contemporary political and legal efforts to restrict and prohibit reproductive healthcare as an issue of primarily philosophical difference obscures and can even legitimize efforts at controlling women, girls, and others capable of pregnancy, by directing the conversation away from these obviously harmful and unjust outcomes, and towards what may seem like more reasonable, and less culpable, differences in abstract views about metaphysics or religion. So, it should not be framed exclusively or even primarily in these terms. At the same time, it is *also* correct to note that the distinction between liberal and illiberal approaches to abortion law plays a significant role in this discourse. Furthermore, this key distinction sheds light on the debate about brain death public policy.

## The Universalist View on Brain Death Policy

One prominent view in brain death policy discourse holds that (i) brain death is death, and (ii) neurologic criteria for death should be legally applied to all: individuals should not be granted exemptions or allowed to opt out from death determination by neurologic criteria (Russell et al. 2019; Omelianchuk and Magnus 2022). The first clause might be defended on several grounds. Some argue that neurologic criteria for death represent a biomedical operationalization of the death of the human organism, that brain death is biological death (e.g., Shemie et al. 2014). Others hold that the biological organism remains alive in brain death, but the person or embodied mind has ceased to exist, and therefore, though not biological death, brain death is death (e.g., Lizza 2006). A third view holds that biology cannot answer the question as to whether brain death is death, however, there are significant moral and social reasons to identify brain death as death, and therefore it is (e.g., Khushf 2010). The second clause generates the name “universalist.”

These different defences of brain death as death are not consistent with each other; the truth of any one entails the falsity of the others. Nevertheless, when combined with the view on public policy that says that neurologic criteria should be applied without exception, each can be a variant of the dominant universalist view that says that brain death is death (for whatever reason), and brain death should be applied to all without exception.

The legal declaration of death has significant consequences. The patient becomes, legally speaking, a decedent, a corpse without legal rights, and no longer an unconscious living patient for whom typical rights and practices such as precedent autonomy and surrogate decision-making would apply. Treatment cessation is mandatory without regard for surrogate decision-making or any applicable advance directives, unless the individual is to be an organ donor. (A court may intercede, but this is not the usual scenario.)

There has been a somewhat exasperating repetition of the claim that there is a mostly settled, worldwide consensus that brain death is death (Wijdicks 2001; Greer et al. 2020). This is false (Yang and Miller 2015; Shewmon 2021). As mentioned above, the “consensus” about brain death, such as it is, is grounded in at least three distinct and mutually inconsistent views about *why* brain death is thought to be death. Nonetheless, they each agree *that* brain death is death, in some sense of the word “death.” By contrast, many conclude that a patient declared “brain-dead” remains alive.

One might argue that brain death is not death on physiological grounds, because the organism continues to function in maintaining homeostasis and resisting entropy (Shewmon 2001; Miller and Truog 2012; Nair-Collins and Miller 2017). Indeed, one of the core views defending brain death, the personhood view, agrees that the body is still-living. One might also bypass any explicit theory about biological death and simply point out that, whatever is the best theory, corpses do not gestate fetuses, grow, or sexually mature, nor manifest greater physiologic stability on home ventilation as compared to another patient who is surely alive but unstable and dying in an Intensive Care Unit (ICU) (Shewmon 2010). Some argue that the case for accepting brain death as death has not been sufficiently established and therefore it should not be accepted (Pellegrino 2008). While some interpretation of most major religions can be found in support of brain death, these interpretations are balanced by doubts about brain death as well, from the perspectives of Buddhism, Shinto, Confucianism, Taoism, Judaism, Catholicism, and Islam (cf. Nair-Collins 2013, 84). And finally, many families, when confronted with the heart-wrenching circumstance of a loved one who has suffered this most unfortunate injury, accept the evidence of their own senses, seeing a deeply comatose but still-living human body, and not a corpse despite what clinicians allege.

Furthermore, if current brain death practices were so settled and well accepted, then “authoritative” statements from medical societies would not need to be released every few years, restating what is allegedly so well-accepted (e.g., Wijdicks et al. 2010; Shemie et al. 2014; Russell et al. 2019; Greer et al. 2020; new guidelines are again in development by the American Academy of Neurology); the U.S. President’s Council on Bioethics (2008) would not have needed to revisit what its predecessor had purportedly already established twenty-seven years earlier (President’s Commission for the Study of Ethical Problems in Medicine and Biomedical and Behavioral Research 1981); and the Uniform Law Commission would not need to consider revising the 1981 Uniform Determination of Death Act in 2022.

Parallels between abortion and brain death are clear. In brain death, there is a class of humans, whose metaphysical and moral status is disputed. Questions are raised involving biological life and death, personhood, the scope and limits of autonomy, and the role of religion, culture, and science. Disagreements about these issues are often rooted in the same sorts of large-scale worldviews that perspectives on abortion are. And the same question arises: What laws and policies would a just society enact?

## Coercion and Illiberalism

Illiberal approaches can be identified with respect to both questions. For abortion, an illiberal political philosophy identifies a substantive answer about the metaphysical and moral questions surrounding a fetus, and seeks to enforce practices that accord with that view while prohibiting those that do not. This is the aim of the pro-life movement: to control all reproductive decisions involving a fetus so that they are made in accord with the movement’s substantive conception of the moral status of the fetus. The result is that women, girls, and others capable of pregnancy will be forced to carry a pregnancy even against their will or if it is harmful to them.

For brain death, an illiberal political philosophy identifies a substantive answer about the metaphysical and moral questions surrounding brain death, and seeks to enforce practices that accord with that view while prohibiting those that do not. Unlike abortion, there is not a single metaphysical conception underlying the view that brain death is death. However, there is one and only one acceptable answer as to *whether* brain death is death: it is. This substantive view on the moral and metaphysical meaning of brain death is held to be uniquely correct and rightly applied to all. Exemptions or opt-outs from death determination by neurologic criteria are sought to be prohibited, in accordance with an illiberal political philosophy.

Notably, one influential proponent of the universalist approach to brain death, the American Academy of Neurology, asserts the right of its member physicians to refuse to participate in brain death determination “based on religious or moral conscience” (Russell et al. 2019, 230), and should transfer the patient to another physician. In the same guidelines, they also state “there is no ethical obligation to provide medical treatment to a deceased person” (though *whether* they are deceased is precisely what is disputed) and endorses unilateral treatment withdrawal over objection (Russell et al. 2019, 231). Hence, the Academy asserts that the deep values of member physicians who oppose brain death should be respected and they should not be coerced into acting in ways not in accordance with them; the values of patients and families should not be respected, even though in this situation the objecting physician and objecting family hold the same view.

The result is that cessation of medical treatment is coercively mandated, requiring death as measured by circulatory criteria, based on a disputed metaphysical view about life, death, personhood, and moral status, detractors to which have at least as much claim to have their views respected as proponents do. No end-of-life healthcare decisions that are in discord with the dominant metaphysical and moral view about brain death are permitted. This is the same coercion about end-of-life healthcare that the pro-life movement seeks to achieve regarding reproductive healthcare.

## Resource Allocation

If treatment removal were not mandatory in brain death, one might worry of a sharp uptick in ICU bed use, potentially to the point that other patients would die because of unavailability of bed space. Therefore—so this objection goes—no brain death exemptions should be honoured, and coerced treatment removal should continue.

Assume that the disastrous outcome postulated by this objection really were to occur. This would not show that the universalist view on brain death is not relevantly similar to the pro-life view on abortion. If the disastrous outcome really were to occur, my point stands.

Additionally, there is no evidence to support the conclusion that the disastrous outcome would occur. In the United States, brain death accounts for 1–2 per cent of all deaths, or about 48–64 incidence per million population. In the European Union it is about 2–3 per cent of annual deaths. In the United Kingdom, it is about 16 per million population, while in Japan incidence drops to 0.25 per million population and 0.02 per million in China (Council of Europe 2013; Yang and Miller 2015; Seifi et al. 2020). There are cultural and historical differences underlying the wide variation observed, but even in nations very acceptive of brain death, it is rare. Some of those declared brain dead go on to become organ donors, so would not receive continued treatment beyond what they receive now. In the United States, more than half become organ donors (Sheehy et al. 2003).

Allowing some people to have exemptions does not imply that others should be coerced into treatment they do not want. Most people would not want extended treatment in this condition, for themselves or their family. Of those who would, systemic derangements that typically accompany the pathophysiologic process associated with brain death are often lethal despite medical intervention. While it is not true that everyone meeting diagnostic criteria for brain death will inevitably suffer cardiovascular collapse in a short time, many will.

Thus, any estimate of increased ICU usage must account for the following: the rare incidence of brain death to begin with, from which those who become organ donors are subtracted. Of the remaining pool, a minority would prefer extended treatment. Of those who prefer extended treatment, a portion, probably a minority, would survive beyond the initial few weeks of the acute phase. Of those who survive, nearly all would be discharged from the acute care hospital, either on home ventilation or to a long-term respiratory care facility. These patients would be expected to return to the acute care hospital more often than an average patient (though not, perhaps, more often than an average patient on long-term ventilation). I don’t have any specific epidemiologic estimates to offer on this very narrow question—nor does anyone else, as far as I know. But we are talking about a very small number of additional patients, a percentage of a percentage of a percentage of a very low incidence of brain death to begin with. This is unlikely to seriously impact the operations of an otherwise reasonably functioning hospital system.

In the United States, New Jersey has had a law exempting individuals from being declared dead by neurological criteria if they had religious objections since 1991. There is no indication that ICUs of New Jersey have been overrun by brain death exemptions. Son and Setta (2018) examined frequency of brain death exemptions and found an estimated 30–36 cases in a five-year period across eighteen hospitals. This provides proof of concept that tolerance of different end-of-life views is unlikely to cause ICUs to swell beyond capacity.

Furthermore, allowing refusals of brain death does not imply that the usual standards for just allocation of resources are inapplicable (Nair-Collins and Hitt 2012). In the case of an ICU that is genuinely confronting a potentially lethal situation of severe resource constraints, it is just to deny ICU admission, move a patient out of the ICU, or even remove ventilation to provide space for another, if this results in an overall more just distribution of scarce resources. The kinds of considerations relevant to a just distribution include, among others, efficacy of treatment and prognosis with and without treatment. But, and crucially, these considerations apply to everyone. *Any* patient with a very poor prognosis could be justly denied space in favour of another patient with a better outlook in a situation of severe scarcity. This has nothing to do with brain death *per se* but with more general considerations that apply to everyone.

I emphasize that these considerations are wholly subsidiary to my key point. There are important parallels between efforts to prohibit abortion and efforts to enact universalist policies on brain death. Both are grounded in coercion and illiberalism. Even if available evidence suggested the disastrous outcome is likely (it does not), this would not obviate the point that the universalist view about brain death is analogous to the pro-life view about abortion.

## Conclusion

There are important similarities between proponents of brain death and opponents of abortion: both advocate coercive control of healthcare decision-making, grounded in an illiberal political philosophy.

The brain death proponent and the pro-life proponent both say:

We know what the uniquely correct answer is regarding the moral and metaphysical status of these classes of individuals, and on that basis we know what behaviours are mandatory or prohibited. If you disagree, you should be approached with kindness and sensitivity (perhaps), but you are nonetheless mistaken. The coercive authority of the State should be used to enforce our metaphysical and moral view onto everyone, whether they agree or not.

This is illiberalism writ large. I would hope that anyone who sees its flaws regarding abortion will apply the same logic to brain death.
